# Diasporas as a linchpin in local and international humanitarian action: a case study of the Chinese in Aceh following the 2004 tsunami

**DOI:** 10.1111/disa.12633

**Published:** 2024-06-18

**Authors:** Miwa Hirono

**Affiliations:** ^1^ College of Global Liberal Arts Ritsumeikan University Japan

**Keywords:** China, contextual memory, diaspora, ethnic groups, humanitarian assistance, international rescue team, localisation, overseas Chinese, social capital

## Abstract

Chinese humanitarian actors have worked frequently with the Chinese diaspora in disaster‐affected areas, but little, if any, research has been conducted into the important role of the diaspora in disaster response and humanitarian assistance. This paper investigates what local knowledge the Chinese diaspora has offered to humanitarian actors from the People's Republic of China (PRC), and how this has contributed to their effectiveness. Based on a case study of the semi‐autonomous Indonesian province of Aceh in the aftermath of the Indian Ocean tsunami of 2004, this paper argues that the diaspora can serve as a linchpin in local and international humanitarian action. It can do so by strengthening networks and bringing together local ethnic communities, local governments, and the PRC's humanitarian actors, while also offering local knowledge in the form of contextual memory. Such local knowledge may have to be fully utilised to address any underlying ethnic tensions in disaster‐affected areas.

## 要旨

中国の人道支援活動家たちは、災害の影響を受けた地域で華僑と頻繁に協力してきたが、災害対応や人道支援における華僑の重要な役割についての研究はほとんど行われていない。この論文は、華僑が中華人民共和国の人道支援活動家たちにどのようなローカルナレッジを提供してきたのか、そしてそれがそれらの活動家の有効性にどのように貢献したかを問うものである。中国の人道支援に焦点を当てた研究は増えているが、被災地における華僑の存在や、それが果たしている役割についてはほとんど注目されていない。この論文では、2004 年の津波後のアチェの事例に基づいて、ネットワークを強化し、地元の民族コミュニティ、地方自治体、中国の人道活動主体を結びつけることによって、華僑が地元および国際的な人道活動の基軸として機能できると論じている。 また、文脈記憶の形でローカルナレッジも提供する。災害の影響を受けた地域の根底にある民族的緊張に対処するには、そのようなローカルナレッジを最大限に活用する必要があるかもしれない。

## 摘要

中国人道主义行动者经常与受灾地区的华侨合作，但对于华侨在灾害应对和人道主义援助中的重要作用的研究却很少。本文探讨海外华侨向中华人民共和国的人道主义行动者提供了哪些本地知识，以及这些知识如何提高这些行动者的效力。越来越多的研究关注中国的人道主义援助，但灾区华侨的存在及其发挥的作用却很少受到关注。本文以 2004 年海啸过后的亚齐为例，指出侨民可以成为当地和国际人道主义行动的关键，通过加强网络，将当地族裔社区、当地政府和 PRC 人道主义行为者聚集在一起，同时以情境记忆的形式提供当地知识。必须充分利用这些当地知识来解决受灾地区潜在的种族紧张关系。

## 1 INTRODUCTION

*After arriving at the destination, we will next tackle the problems of language, transportation, local guides, etc. During previous overseas rescues, overseas Chinese have been particularly helpful* (Xinjing Bao, 2023).^1^

Within 48 hours of the 7.8‐magnitude earthquake that struck Turkey and Syria on 6 February 2023, more than 100 members of the Chinese Guizhou Blue Sky Rescue Team gathered together in Guiyang Longdongbao International Airport to fly to Turkey to conduct rescue operations. The above quote was by Wang Yi, the Captain of the Guizhou Blue Sky Rescue Team, before his departure from Guizhou. He further suggested that after the Chinese team discussed the relief process that would unfold under the guidance of the relevant United Nations (UN) coordination mechanism, the team would ‘get in touch with overseas Chinese organisations’ in Turkey, ‘which will also actively participate in the rescue work’ (Xinjing Bao, 2023).

Ever since China established its international rescue team in 2001, one of the major problems faced in rescue operations has been the language barrier. In emergency rescue operations, having smooth communication is essential. While the Chinese rescue team, often consisting of People's Liberation Army officers, seismic specialists, and medical doctors, has confronted some language difficulties (Hirono, 2018, p. 27), China has a substantial ‘local resource’ in many countries: overseas Chinese people. They frequently have bilingual or multilingual skills—that is, speaking Chinese and one or more local languages.

However, the role of overseas Chinese—the Chinese diaspora—brings into relief a very tricky question for political, economic, and historical reasons. Politically, it is a diaspora that relates to China, a country that often attracts significant negative attention, in the form of scepticism and perceptions of it presenting some sort of threat. In the context of President Xi Jinping's regime, ‘infiltration’ by China into the societies of other countries is a politically sensitive issue across the world (Meyers, 2018; Cunningham, 2022). The Chinese government and the Communist Party of China (CPC) bolster and incorporate their overseas Chinese networks into ‘a strong force to work together for national rejuvenation’, as Xi Jinping claimed in his report made on behalf of the nineteenth Central Committee of the CPC, delivered at the twentieth National Congress of the People's Republic of China (PRC) (The National People's Congress of the People's Republic of China, 2022). Economically, China has the potential to provide significant assistance and economic investment after a disaster to support the process of reconstruction. Historically, as some Southeast Asian states' anti‐China sentiments demonstrate, there is a degree of ethnic animosity towards overseas Chinese in some countries. With all these complications, to believe that China's international rescue team simply teams up with local overseas Chinese as ‘language, transportation, and local guides’ for a smooth operation is naive.

This paper is not about the Chinese government's political motives for working with the Chinese diaspora. Rather, it is about local perspectives—the perspectives of the overseas Chinese as ‘the local’ in disaster‐affected communities. Who is ‘local’ is a difficult question, of course. It is often a subjective concept. However, the fact that many overseas Chinese have lived in a local area for more than several decades and that they recognise themselves as ‘Chinese Indonesians’, for example, means that their identity should be regarded as that of ‘the local’ apropos of their contribution of local knowledge to disaster relief and humanitarian aid operations—regardless of the fact that a majority group might refer to ethnic minority groups as ‘non‐local’. Minority groups have indispensable skills and local knowledge to offer to disaster management at the local level.

Overseas Chinese have so much local knowledge in the form of contextual memory. Their living historical memory of anti‐China movements in the countries in which they reside—the extent of which has waxed and waned from generation to generation—constitutes a very important context in which disaster responses take place. Having contextual memory is essential to an effective operation because it gives ‘meaning’ to a particular action. If the Chinese rescue teams accord too much priority to rescuing local overseas Chinese, this may be interpreted as unbalanced, unfair, and indeed too much intervention. In contrast, if local overseas Chinese can play a vital role in identifying what action is needed in a particular local area in a way that addresses the relevant contextual memory, then Chinese rescue operations are likely to be welcomed—one effect of which may be to raise the social status of the overseas Chinese as well. In this respect, clearly, overseas Chinese support provided to the Chinese international rescue team goes beyond the ‘language, transportation, and local guides’ mantle and extends to the sharing of information about what kind of operation is regarded as ‘appropriate’ by the majority group of the society.

Against this background, this paper asks two questions: (i) what local knowledge did the Chinese diaspora offer to the PRC's humanitarian actors in the semi‐autonomous Indonesian province of Aceh in the aftermath of the Indian Ocean tsunami of 2004?; and (ii) how did local knowledge help make China's humanitarian assistance effective? The literature on China's humanitarian assistance tends to focus on its political intentions and its geographical expansion (Tubilewicz, 2012; Gong, 2021), but little, if any, research has examined how China's international rescue team operates on the ground, particularly in relation to its localisation endeavours. When a disaster hits a multi‐ethnic community, a diaspora can play a pivotal role. A diaspora and ethnic groups can be a linchpin that holds local and international efforts together, strengthening networks and aiding the coordination of local ethnic minorities, local governments, and international rescue teams, based on local contextual memory.

This paper answers these two questions using a case study of the role of the Chinese diaspora in Aceh following the Indian Ocean tsunami on 26 December 2004. The reason for this example is that the Chinese diaspora has experienced significant enmity in the past from the majority population in Aceh. In fact, during violence in 1965–66, the Chinese in Aceh suffered what some writers describe as an outcome bordering on genocide (Melvin, 2013).

This is an intricate case study, therefore. The Chinese diaspora was able to help bring together Chinese networks in Aceh to support the Chinese international rescue operation, but, potentially, it could also have engendered ethnic tension between the Chinese and the majority Acehnese. Given the situation, the case study has significant implications for the role of an ethnic minority in disaster response. Moreover, we could also compare this historical example with more recent cases where the Chinese rescue team and the Chinese government have developed a more systematic approach to establishing links with overseas Chinese, but that is beyond the scope of this particular paper.

For a total of three weeks in October 2009, I undertook a total of 30 semi‐structured interviews with a wide range of local Acehnese people and people who worked in Aceh and Jakarta, the capital of Indonesia, including Acehnese academics, international academics, local government officials, international aid workers, Acehnese medical workers, overseas Chinese villagers who reside in the Indonesia–China Friendship Village (known locally among the Acehnese as the ‘Jackie Chan Village’), one ethnic Chinese person in Aceh, and one Chinese diplomat. In addition, I visited the Friendship Village, which is a signature project of the Chinese government and the Chinese Red Cross (as discussed below), where interviews with overseas Chinese villagers took place. The interviews covered the emergency relief period from December 2004 to March 2005, as well as the reconstruction period from April 2005 to April 2009, using the periodisation of the Indonesian government.^2^

The paper is divided into three sections. The first section engages in a theoretical discussion of the role of a diaspora in disaster response and identifies contextual memory and social capital as the key local knowledge that a diaspora offers. The second section provides an historical overview of how the Chinese international rescue team has worked with local Chinese diasporas and locates the case of Aceh within the 20‐year history (2003–23) of China's rescue operations. Its aim is to pinpoint the various ways that ‘local’ overseas Chinese have operated to support various Chinese international rescue teams in disasters and to determine the nature of those instances in relation to the case of Aceh. The third section assesses the case of Aceh and argues that the local knowledge of contextual memory of overseas Chinese people in the semi‐autonomous province, as well as social capital, actually mattered in terms of the success of the operations. Furthermore, it contends that making use of such local knowledge helped China's humanitarian assistance operations to overcome the language barrier, facilitate needs analysis, coordinate operations, and address social stigma.

This paper asserts that the diaspora served as a linchpin in local and humanitarian action not only by strengthening networks and bringing together local ethnic communities, local government, and the PRC's humanitarian actors, but also by offering local knowledge in the form of contextual memory. Such local knowledge has to be fully applied to address underlying ethnic tensions in disaster‐affected areas. Even though it may not have been the PRC government's intention, the effective contribution of the local Chinese diaspora in Aceh in 2004 added a dimension to the debate on the importance of local knowledge in disaster response.

## 2 ROLES OF A DIASPORA IN HUMANITARIAN RESPONSE AND CONTEXTUAL MEMORY

Diasporas are key local actors in a humanitarian emergency. Leading international organisations and thinktanks that deal with humanitarian issues have begun to focus on the importance of local diasporas and to explore ways in which to include them in the existing humanitarian coordination mechanism (iDiaspora, n.d.). Despite the significant and unique role that a diaspora can play in disaster response, however, international policy discourse does not pay much attention to a diaspora as a local actor, let alone as a source of local knowledge to be used in disaster response. For example, the International Organization for Migration has implemented a project to develop a ‘framework for diaspora engagement in humanitarian assistance’ (International Organization for Migration, 2021). Diaspora Action Australia (Phillips, 2018, p.12) has also looked at diaspora roles in times of disaster, suggesting that ‘diaspora organisations be encouraged to register locally, for example with UNOCHA [United Nations Office for the Coordination of Humanitarian Affairs] and NGO [non‐governmental organisation] Forums, so they can be part of existing coordination structures’ (Phillips, 2018, p. 12).

Diaspora Emergency Action & Coordination (2022) has put forward three models, offering a useful categorisation of how the local knowledge of a diaspora can be made manifest in a disaster response. Each of the models corresponds with each of the three types of social capital: bridging, bonding, and linking (Aldrich, 2012; Szreter and Woolcook, 2003). The first model is a partner model, in which ‘[d]iaspora organizations implement responses with local partners, mainly L/NNGOs [local and national non‐governmental organisations] and community‐based organizations’ (Diaspora Emergency Action & Coordination, 2022, p. 12)—this relates to the ‘bridging’ form of social capital between communities. The second model is a direct model, in which ‘[d]iaspora organizations implement responses directly with their own staff and/or volunteers’ (Diaspora Emergency Action & Coordination, 2022, p. 13)—this relates to the ‘bonding’ form of social capital within a community. The third model is a sub‐grantee model, in which ‘diaspora organizations carry out responses as implementing partners for UN agencies and/or INGOs [international non‐governmental organisations]’ (Diaspora Emergency Action & Coordination, 2022, p. 13)—this relates to the ‘linking’ form of social capital.

While these studies are an important development, they often ignore the historical processes of some diaspora groups: various diasporas experience occasional prejudice and discrimination. In 1923, for example, a massacre of 6,000 Koreans, and 700 Chinese who were mistaken for Koreans, in the aftermath of the Great Kanto Earthquake was caused by rumours that Koreans had poisoned local wells and planned violent attacks against Japanese citizens (Morris‐Suzuki, 2021). Today, a significant impact of racial and ethnic disparity and discrimination, or fear thereof, on disaster responses is reported in many parts of the world (see, for example, Fothergill, Maestas, and Darlington, 1999). Emerging research and policy interest in the roles of diasporas in disasters must bring to the fore local knowledge, such as their contextual memory, and address any of their problems, fears, and concerns.

## 3 CHINESE INTERNATIONAL RESCUE TEAMS AND THE CHINESE DIASPORA

How have China's international rescue teams engaged with the Chinese diaspora? This section reviews the evolution of the cooperative relationship between China's international rescue teams and the various Chinese local diasporas since the first major operations at the beginning of the twenty‐first century. As demonstrated below, the evolution has the following three features:

- First, Chinese local diasporas have consistently offered language and logistical support to China's international rescue teams and other humanitarian organisations.
- Second, local diaspora support has developed from sporadic individual efforts to more collaborative organisational initiatives, relating to the PRC's intention to involve the diaspora in disaster response.
- Third, an increasing number of Chinese business groups—mostly composed of members of the local diaspora and even some transnational businesspeople—have undertaken to support the PRC's disaster responses.

The China International Search and Rescue (CISAR) team (Zhongguo guoji jiuyuandui) was established in April 2001, and its first major operation was in Aceh in December 2004—the case study of this paper. As detailed below, the Chinese local diaspora in Aceh was quite helpful in assisting the CISAR team by providing language support. The Chinese local diaspora volunteers offered ‘assistance to the team in terms of interpreters, guides and material procurement’ (Zhongguo Xinwen Wang, 2006). Furthermore, the local diaspora collected and donated approximately USD 6,000 of medical supplies, such as plaster bandages and internal fixation plates, which were urgently needed by the rescue team, erected three awnings spanning a total area of about 100 square metres at the rescue team's camp, and donated a large number of daily necessities to the rescue team (Xinhua Wang, 2006). While Indonesian President Susilo Bambang Yudhoyono expressed his gratitude to the CISAR team in 2006 (Zhongguo Xinwen Wang, 2006), the support of the Chinese local diaspora lay behind the successful operation.

At the time of the Great East Japan Earthquake in March 2011, however, the Chinese rescue team's activity was rather limited owing to the Japanese perception of a threat to its security implicit in the CISAR team's use of the United States' military base in Misawa, Aomori Prefecture (Hirono, 2011). The Chinese government reported that the CISAR team ‘had sent word from the front that 18 Chinese nationals in Japan, who are unable to contact their families at home, as well as 68 Chinese from five branches of Xinhua International Corporation in Ofunato City, Iwate Prefecture, wished to report their safety to those at home in China through the rescue team’ (Zhongyang Zhengfu Menhu, 2011).

The Gorkha earthquake in Nepal in April 2015 resulted in a major operation by the CISAR team. A new development at that time was that a non‐governmental team called the Blue Sky Rescue Team joined the main Chinese government force in conducting operations. Nepal did not have then, and nor does it have now, a large Chinese local diaspora. In the capital, Kathmandu, it was a single Chinese trader in the country who supported the operation by providing mainly food and logistical support, such as accommodation. This may have significant implications for the PRC's future humanitarian assistance, given expanding trade activity under the Belt and Road Initiative. The ‘Chinese diaspora’ no longer consists of only those who have lived in the local area for more than a decade, but also now includes local Chinese traders and Chinese economic actors who have resided there for a shorter period (Fenghuang Weishi, 2015).

Then came even more and better coordinated Chinese local diaspora support for Chinese rescue operations in Attapeu Province, Laos, in July 2018, in the aftermath of flash flooding caused by a breach of the Xe Pian‐Xe Namnoy hydropower saddle dams (Haiwai Net, 2018). When the Chinese Blue Sky Rescue Team, consisting of 76 volunteers, arrived in the capital, Vientiane, they met with members of the Laos Vientiane Chinese Help Centre and a number of Lao Chinese volunteers to go to the disaster area. Afterwards, the rescue team held a joint coordination meeting with the Laos Vientiane Chinese Help Centre. This was followed by discussions involving the head of the Blue Sky Rescue Team, the Lao‐Chinese Friendship Association, the Vientiane Branch of the Industrial and Commercial Bank of China, and the Lao Chinese Chamber of Commerce, the Hunan Chamber of Commerce, and the Guangdong Chambers of Commerce, on the topic of routes to the disaster area, the deployment of rescue equipment and materials, the allocation of rescue personnel and vehicles, accompanying interpreters, and other matters. As a result of these talks, the Blue Sky Rescue Team gained an understanding of the current situation in Attapeu Province, and of Lao customs and traditions, and formulated a rescue plan (Haiwai Net, 2018). As a side note, how humanitarian and economic motives intersect in Chinese local diaspora support are beyond the scope of this paper but are worthy of further exploration and analysis.

The Laos Vientiane Chinese Help Centre is one of many ‘Chinese Help Centres’ (Huazhu Zhongxin) that the State Council's Overseas Chinese Affairs Office established in a number of Southeast Asian countries on 9 March 2014—the State Council is the chief administrative authority of the PRC (Renmin Wang, 2014). The centres have a mandate to ‘offer care and help, emergency handling and other related services for overseas Chinese’ (Chuguo Dagong Jiuye, 2022). They have played a pivotal role in bringing into being many organisations set up by the Chinese diaspora to support rescue operations. The centres may facilitate even more consolidated diaspora support for the Chinese international rescue team in the future, making the role of the Chinese diaspora in disaster response all the more important.

In addition, in Phuket, Thailand, after a storm capsized and sank tourist boats in July 2018, resulting in 46 deaths and three missing persons, the Southern Thailand Chinese Help Centre played an important part in coordinating volunteers, more than 70 per cent of whom were Chinese. Wei Guanglei, the Secretary‐General of the Centre, said that ‘the number of volunteers involved in the rescue of the accident was beyond expectation’ (Renmin Wang, 2018), suggesting that the Centre acts as a hub to facilitate participation by many overseas Chinese in volunteer rescue operations.

The earthquake in Turkey in 2023 also witnessed concerted efforts by overseas Chinese people to support Chinese rescue teams, although the country does not have a government‐built Chinese Help Centre. The Chinese Blue Sky Rescue Team met with a number of local Chinese organisations and contacted local Chinese volunteers in Turkey (Xinjing Bao, 2023). As soon as its members arrived at Malatya Erhac Airport, the Chinese local diaspora picked them up and ‘acted as an accompanying interpreter to help the rescue team with language, transport and guide issues, and the team was transported to a local school for assembly’ (Peng, 2023). Next the team met with the Chinese Chamber of Commerce and Industry, private associations, and international students in Turkey, which together comprise the local ‘overseas Chinese network’ (Zhongguo Qiaowang, 2023). After their arrival, too, ‘the local Chinese volunteers and Chinese‐funded enterprises became an important communication bridge between Chinese rescue forces and the earthquake‐stricken areas in Turkey’ (Zhongguo Qiaowang, 2023).

In all of these disaster‐affected regions, the Chinese local diasporas have their own contextual memory situated within the locality in which they have lived. In addition, in the Xi Jinping presidential period (since 2013), the Chinese diaspora has often experienced the downside of a reputation of having some sort of ‘link’ or association with a PRC that has a proactive foreign policy posture. No matter how substantial or nominal such a ‘link’ may be, China under Xi adds a further complication to the study of Chinese local diasporas, because of the need to address both anti‐China sentiment in the historical period and the perception of a ‘China threat’ in today's world. While any study of the relationship between the Chinese diaspora and the PRC with regard to disaster response in the Xi Jinping period must treat the two (the diaspora and the PRC) as integral, it would be helpful to isolate the diaspora from the impact of the PRC, by studying a pre‐Xi Chinese local diaspora, such as in the aftermath of the Indian Ocean tsunami of 2004, well before China assumed its current proactive foreign policy posture under Xi. This way, one can focus on the local knowledge offered by the Chinese local diaspora to the Chinese international rescue team, without having to consider Xi's role or the PRC's influence on the diaspora community.

## 4 CHINA'S ASSISTANCE IN ACEH

Aceh suffered a 9.0‐magnitude earthquake followed by a devastating tsunami on 26 December 2004. The tsunami alone claimed more than 200,000 lives and destroyed 141,000 houses (Oxfam, 2006). The international humanitarian emergency assistance response was the largest in history. Reconstruction efforts were among ‘the largest … in the developing world’, with a total of USD 7.7 billion committed by the Indonesian government and the international community (World Bank, 2008). China's humanitarian post‐disaster assistance of USD 63.57 million was the sixteenth largest commitment to Aceh in comparison with other contributing states.^3^ In China's history of humanitarian assistance, this was by far the biggest financial contribution until it offered USD 125 million to West Africa to combat the Ebola Virus Disease in 2014. The sum dispersed to Aceh comprised USD 33.51 million from the Chinese government and USD 30.06 million from so‐called government‐organised NGOs (non‐governmental organisations), such as the China Charity Foundation and the Red Cross Society of China. The large sum of money supported primarily the following five major projects:

1. Four batches of emergency rescue teams, involving 115 personnel in total (USD 8.19 million) (Li et al., 2005, p. 272; Renmin Wang, 2005; Zhongxin Wang, 2005).^4^
2. Sixty prefabricated houses in Aceh (USD 7.23 million).^5^
3. Two hundred containers of student stationery and school bags (USD 5.5 million) (Zhongguo Waijiaobu, 2006).^6^
4. Donation of funds (USD 5.5 million).^7^
5a. The building of the Indonesia–China Friendship Village (USD 8 million).^8^
5b. The building of the Indonesia–China Friendship Village (USD 3.4 million).^9^

Projects 1–3 were by the Chinese government, whereas projects 4 and 5a were by the China Charity Foundation and 5b was by the Red Cross Society of China.

Of these projects, this paper pays particular attention to two representative elements of China's post‐disaster assistance: the Chinese government's emergency rescue work conducted from December 2004 to February 2005 (projects 1 and 2); and the building of the Indonesia–China Friendship Village (hereafter the ‘Friendship Village’), implemented from October 2005 to July 2007 (project 5 (a and b)). The latter is a signature project of the China Charity Foundation and the Red Cross Society of China, as discussed below.

China's assistance to Aceh was politically sensitive for two reasons. The first centred on the state of Sino‐Indonesian relations. The bilateral relationship between the two countries had been normalised in 1999, only five years before the tsunami. Since the 1960s, Indonesia had been very concerned about what it perceived as the ‘China threat’—or more precisely, the ‘triangular threat’ posed by the CPC, the Indonesian Communist Party, and the ethnic Chinese in Indonesia (Sukma, 2009). Furthermore, there had been, and to some extent there remains, deep‐rooted local suspicion of the ethnic Chinese in Indonesia.^10^ The second reason pertains to Jakarta–Aceh relations. Aceh had been fighting for independence since 1976. From the Indonesian government's perspective, the decision to allow foreign military personnel and foreign aid workers into the province was nerve‐wracking. So much so that the Indonesian government sent out an early warning to foreigners to withdraw from Aceh as soon as the humanitarian assistance work there had been completed (Perlez, 2005).

In such a politically sensitive local context, what roles did the Chinese diaspora play? The following section examines how its roles evolved as the emergency response and reconstruction periods unfolded in Aceh. It does so by making reference to the aforementioned three roles diasporas can play—those of partner, direct, and sub‐grantee—and the type of social capital enhanced by the diaspora during their disaster response.

## 5 ROLES OF THE DIASPORA

### 5.1 Social capital within the Chinese community

When the tsunami hit Banda Aceh, the capital of Aceh, most of the 5,000 Chinese people who lived there evacuated to Medan (a city located 600 kilometres from Banda Aceh), which has a higher concentration of Chinese Indonesians than Banda Aceh. More than 60 local Chinese communities in Medan formed a disaster relief committee to provide the evacuees from Banda Aceh with free accommodation and medical treatment. However, confronted by more than 3,000 Chinese Acehnese subsequently returning from Medan to Aceh, the Acehnese government allowed them to establish their own social organisation, called the Aceh Chinese Charity Foundation (Huanqiu Shibao, 2005).

This would have been impossible before the earthquake owing to political sensitivity regarding the existence of the Chinese in Aceh. Local suspicion towards ethnic Chinese was particularly strong in the province. The ‘China Town’ in Aceh did not show any written Chinese characters. The celebration of Chinese New Year on the street was banned nationally until 2001. Creating a Chinese social organisation was not even conceivable in Aceh, but the tsunami and the resulting disaster response operations changed the political landscape.

The Aceh Chinese Charity Foundation continued to work on behalf of tsunami victims, focusing on the widows and orphans among the Chinese victims in Aceh (Huanqiu Shibao, 2005). In the first spring festival after the tsunami, the local Acehnese Chinese gathered together at a temple on higher ground, which was less damaged and from which debris had been cleared. ‘The temple has become a symbol of refuge and self‐sufficiency for the local Chinese. Many Chinese from Aceh province [actively] rely on assistance from Medan and other Chinese communities rather than [passively] enjoying the assistance given by the government and UN relief agencies’ (Shi, 2005). Chen Songmao, the President of the Meide Mutual Aid Association, stated that the tsunami united all overseas Chinese in a fight against disaster (Xinhua News Agency, 2005). Furthermore, in 2006, the Indonesian parliament passed a new nationality law, which enables ethnic Chinese in their local diasporas to participate in local and national elections. In addition, Chinese ethnic groups are more actively involved in post‐disaster relief and reconstruction work in Indonesia through their association (Zhongguo Maoyi Bao, 2013).

In short, of the three models mentioned earlier—partner, direct, and sub‐grantee—the Chinese local diaspora in Aceh managed to apply the ‘direct model’. Diaspora organisations implemented responses directly in their own Chinese community, while creating and enhancing Chinese social capital by ‘bonding’ the local Chinese community in Aceh—a bond that was not allowed to exist in the Indonesian political context. This was the social setting in Aceh that greeted the arrival of the CISAR team.

### 5.2 Language and logistics support

Lao Cangmin, an overseas Chinese person who was born in Aceh after his father emigrated to Indonesia at an early age, was running a photocopy and stationery shop in the province. When the tsunami hit, all members of his sister's and brother's families were killed, and the water flooded his store. On the day after the tsunami, Lao had to evacuate from Aceh to Medan with other Chinese (Souhu Xinwen, 2005). In Medan, however, Lao learnt that the CISAR team was in urgent need of language support. Many Chinese rescue team members could not communicate in English, which made it difficult to coordinate international efforts because the lingua franca of the rescue effort was English.^11^

Lao immediately took the first flight to the Banda Aceh military airport where the CISAR team was stationed (Souhu Xinwen, 2005). Without even going back to the flooded shop, he began working for the team. He said in a Chinese media interview on 14 January 2005 (in the midst of the rescue operations): ‘I came to do volunteer work without thinking there would be any benefit; just wanted to do something useful’ (Souhu Xinwen, 2005).

Lao volunteered to interpret for Chinese medical doctors and local Acehnese patients. He was the only local interpreter working for the Chinese rescue team; he became an interpreter out of a ‘sense of duty’. This needs to be understood in the context of the anti‐communist violence of 1966—what is often called ‘the Exclusion of China’—which led all Chinese to flee from Indonesia and to go back to China. Lao stated that he felt his help could eventually improve the image of China.^12^

A Chinese paramilitary newspaper published an article, titled ‘Lao Cangmin and the armed forces general hospital's “life and death relationship”’, describing him as follows:

*Every member of the Chinese international rescue team knows this enthusiastic local Chinese man. In a moment of crisis, he stepped forward to go to the disaster area in Banda Aceh, volunteering to help the rescue team as a volunteer interpreter, providing selfless help, for the smooth implementation of the work of the Chinese rescue team, making a positive contribution* (Zhongguo Junwang, 2015).

Although he helped the relief workers, Lao's house was still filled with flooded water, which had merged with sewage. His family had gone to Surabaya (a city on the island of Java) following the birth of his grandson, so he was the only one in the family in Aceh. Even though the sewage had built up, Lao thought it was more important to help the relief workers than attend to his own house. The Chinese relief workers were moved to hear this and came to help him clean his house.

Through these interactions, Lao applied the ‘sub‐grantee’ model to his work, via which he became a linchpin between the Chinese rescue team and the local Acehnese community that needed help. Lao's work was entirely on a voluntary basis, without any ‘grant’ as such, but his work certainly helped to generate a ‘linking’ form of social capital, which was used effectively by the Chinese rescue team.

### 5.3 Coordination

Lao's work was not confined to language and logistics support; it also came to include needs analysis and complex coordination. Lao was coordinating with the local Acehnese government to find areas in need of rescue. He then arranged the rescue team's work. This illustrates another ‘linking’ form of social capital that Lao generated between the Chinese rescue team on the one hand, and the local Acehnese government and the main hospital in Banda Aceh on the other.

Humanitarian coordination in the aftermath of the tsunami is notorious. In the early emergency phase, both national and multilateral coordination mechanisms were organised inefficiently. In terms of a national coordination mechanism, the Indonesian government used its own coordination agency, the National Coordinating Body for Disaster Management (Badan Koordinasi Nasional Penanggulangan Bencana dan Penanganan Pengungsi, BAKORNAS), as well as the provincial disaster management office (Satuan Koordinasi Pelaksana Penanggulangan Bencana dan Penanganan Pengungsi, Satkorlak) (Wiharta et al., 2008, p. 89). A Government of Indonesia and United Nations (2005, p. viii) joint report suggests that ‘[p]olicy, structure and mechanisms of BAKORNAS did not function well’, and that it was not until the second week after the disaster that provincial level coordination took effect. At the same time, a multilateral approach also suffered significantly the problem of ineffectiveness. Although coordination meetings are essential in helping agencies to target assistance without duplication, an evaluation of the coordination process in the aftermath of the tsunami claims that ‘[t]he significant opportunity costs of attendance, especially for smaller agencies, outweighed the benefit, particularly when meeting themes and decisions were repeated several times’ (Bennett et al., 2006, p. 10). The diversity and sheer number of government and non‐government agencies participating in post‐disaster assistance led to extreme complexity and severe competition in the implementation of various projects and levels of assistance (Schulze, 2005). As a matter of fact, the number of coordination agencies that emerged in Aceh resulted in confusion and frustration among aid workers on the ground. According to NGO worker Carsten Völz (2005, p. 26), ‘[w]ith more than a dozen UN agencies in Banda Aceh competing for their turf, coordination went into overdrive, with 72 coordination meetings per week in Banda Aceh alone’. Furthermore, ‘[s]imilar UN‐led coordination groups were working on the same issues in Jakarta, Medan, Banda Aceh and Meulaboh, with little communication between them’ (Völz, 2005, p. 26).

However, the CISAR team did not have much engagement with the United Nations Office for the Coordination of Humanitarian Affairs (UN OCHA) and other UN agencies during coordination and project implementation processes. Instead, it adopted a bilateral approach to post‐disaster assistance. In terms of coordination, CISAR team members attended the Aceh provincial government's Coordination and Implementation Unit (Satkorlak) meetings every morning during the emergency phase from late December 2004 to the end of March 2005.^13^

Satkorlak suggested that Chinese medical staff go to Zainal Abidin Hospital (the main hospital in Banda Aceh)—where Lao Cangmin coordinated with doctors to identify specific work for CISAR members. My interviews with the chief doctor and the chief nurse at that hospital, who worked side‐by‐side with the Chinese rescue team members, revealed that the Chinese medical team arrived at the hospital early and worked alongside other medical staff very efficiently.^14^ This was partly because of Lao's contribution. My interviews with international aid workers in Aceh, and with local Acehnese people, suggest that China's rescue efforts were perceived very positively, and that the rescue team's skills and discipline were perceived as being of a high standard.

### 5.4 Needs analysis

The Chinese local diaspora further enhanced the linking form of social capital in the reconstruction phase, but this time, its contextual memory in Aceh was the key foundation on which to build social capital. As far as my interviews indicate, China's reconstruction effort, which concentrated on the building of the Friendship Village, was perceived locally as efficient, thanks to the local Indonesian‐Chinese network.^15^ The latter helped by acting as a liaison between ethnic Indonesian‐Chinese people who needed relocation, and the provincial government, which dealt with its Chinese counterparts on the Friendship Village project.^16^ This was in contrast to reports that many donors experienced problems of efficiency in the reconstruction phase, largely because of ‘the uncritical and uninformed allocation of funds between agencies’ (Flint and Goyder, 2006, p. 37).

How to distribute the 606 houses built in the Friendship Village to victims was a challenging decision. Each neighbouring village submitted information to the Aceh provincial government about who needed housing. The provincial government then asked the Aceh Chinese Charity Foundation to decide who would receive it. Lao Cangmin, in charge of the Foundation's financial matters, conducted a needs analysis. After applicants submitted information on their family and housing situation, Lao and other Foundation staff carried out a visit to investigate whether the family, irrespective of whether they are Chinese or Acehnese, met the condition of financial difficulty, after which housing in the Friendship Village was determined in a lottery (Li et al., 2016).

Throughout this process, Lao not only maintained relations with the Chinese family who needed support, but also with the Chinese Consulate‐General in Medan and with the Acehnese provincial government. These relations helped the Chinese even after reconstruction and rehousing had been completed, because ‘whenever problems arise in the Village, the Village people seem to prefer to go to the Chinese Consulate with Lao Cangmin to solve them’ (Li, 2016). They felt that the local Acehnese government was not always efficient in resolving various issues.

This process required local knowledge of contextual memory regarding the local history of ethnic relations. This is because it is essential to pay appropriate attention to the intricate balance between the majority Acehnese and the local Chinese diaspora. Any disproportionate allocation of housing or other reconstruction support to the Chinese in Aceh, even though funding might have come from the PRC, might have made the reconstruction process impossible. The contextual memory of Lao and his colleagues at the Aceh Chinese Charity Foundation were indispensable to the smooth process of reconstruction.

## 6 CONCLUSION

This paper has examined what local knowledge the Chinese diaspora offered to humanitarian actors from the PRC, and how it contributed to their effectiveness. Based on an examination of the roles that the Chinese local diaspora played in the post‐tsunami emergency and reconstruction process in Aceh, this paper reaches three key findings:

- First, the urgent need for help catalysed the efforts of the Chinese local diaspora to go beyond the pre‐tsunami suppressive relationship with the Acehnese government, to generate social capital within the Chinese community, as well as between the Chinese local diaspora and the PRC, and between the Chinese international rescue team and the Acehnese government and community. In other words, the diaspora served as a linchpin in local and international humanitarian action.
- Second, contextual memory of discrimination of the Chinese in Aceh, the quest to seek approval from the majority Acehnese, and the desire to make some connection with the PRC together encouraged the Chinese diaspora to engage in rescue and reconstruction operations and to offer language, logistics, and coordination support to the PRC's humanitarian actors.
- Third, contextual memory is not just a background to explain the diaspora's wish to help; it also adds invaluable capacity to address the needs of the community while striking the right balance in relation to the majority Acehnese. Contextual memory laid the foundation for the enhancement of social capital, and for the efficient coordination of aid distribution between the majority group and the overseas Chinese, facilitating the PRC's rescue and reconstruction operations.

The implication of this study is significant for future humanitarian assistance, given China's increasing amount of humanitarian assistance and its bolstered efforts to coordinate with, or utilise, the Chinese diaspora. The status of overseas Chinese varies depending on the contextual memory in the society where they reside. The evolution of the relationship between the Chinese local diaspora and the PRC's humanitarian actors demonstrates that the local knowledge of and support from the diaspora are indispensable to humanitarian operations, especially those conducted by the PRC. However, local diaspora support could be a double‐edged sword. To walk the fine line between the wealth of skills and knowledge useful to a rescue and reconstruction operation and historically complex social relations and the more recent sense of a widening China threat in many parts of society, disaster respondents will need to pay very careful attention to the role of the local diaspora and its local knowledge.

## ETHICS STATEMENT

This paper reports analysis of primary data. Persons from whom data were collected gave their free, prior, and informed consent. Data have been kept confidential and used anonymously, unless explicit consent was given to reveal their identity.

## Data Availability

The data that support the findings of this study are available from the corresponding author upon reasonable request.

## ENDNOTES

^1^
All translations in this paper are the author's.

^2^
One should note that there is a debate on the problem associated with separating these two periods (Hilhorst, 2007). Critics argue that administrative separation of them may lead to a situation in which the continuing need for emergency relief tends to get squeezed out when reconstruction takes over. This paper appreciates this evaluation, but for the sake of convenience, it uses the Indonesian government's periodisation.

^3^
Interview with representatives of the Embassy of the People's Republic of China in the Republic of Indonesia, Jakarta, 27 October 2009.

^4^
Additional source is an unpublished internal document of the BRR (Badan Rehabilitasi dan Rekonstruksi) showing funding items not yet incorporated in the Recovery Aceh Nias (RAN) Database generated on 16 October 2009.

^5^
Source is an unpublished internal document of the BRR showing funding items not yet incorporated in the RAN Database generated on 16 October 2009.

^6^
Additional source is an unpublished internal document of the BRR showing funding items not yet incorporated in the RAN Database generated on 16 October 2009.

^7^
Source is an unpublished internal document of the BRR showing funding items not yet incorporated in the RAN Database, 16 October 2009.

^8^
Source is the RAN Database generated on 16 October 2009.

^9^
Source is the RAN Database generated on 16 October 2009.

^10^
Interviews with Aceh‐based academics, Banda Aceh, 13, 16, and 17 October 2009.

^11^
Interview with a medical doctor at Zainal Abidin Hospital, Banda Aceh, 23 October 2009.

^12^
Interview with Lao Cangmin, Banda Aceh, 23 October 2009.

^13^
Interviews with Acehnese government officials working for the emergency response in 2004–05, Banda Aceh, 19 and 20 October 2009. There is little information available on whether the Chinese rescue team attended the meetings organised by multilateral coordination bodies, such as UN OCHA. I asked a number of local Acehnese and those who still work for UN agencies in Aceh, but none of them were aware of a Chinese presence in UN OCHA meetings.

^14^
Interviews with a chief nurse and a chief medical doctor at Zainal Abidin Hospital, Banda Aceh, 22 and 23 October 2009.

^15^
Interviews with Acehnese government officials working for the emergency response in 2004–05, Banda Aceh, 19 and 20 October 2009; as well as with several villagers in the Indonesia–China Friendship Village, Banda Aceh, 23 October 2009, and with Lao Cangmin, Banda Aceh, 23 October 2009.

^16^
Interviews with several villagers in the Indonesia–China Friendship Village, Banda Aceh, 23 October 2009.
